# Revisiting the determination of hydromechanical stresses encountered by microcarriers in stem cell culture bioreactors

**DOI:** 10.1186/1753-6561-9-S9-P41

**Published:** 2015-12-14

**Authors:** Angélique Delafosse, Marie-Laure Collignon, Annie Marc, Dominique Toye, Eric Olmos

**Affiliations:** 1 Laboratoire Réactions et Génie des Procédés, UMR 7274 CNRS - Université de Lorraine, 2 avenue Forêt de Haye, TSA 40602, 54518 Vandœuvre-lès-Nancy, France; 2 Department of Chemical Engineering, Université de Liège, 3, Allée de la chimie, batB6C B-4000 Liège, Belgium; 3 FRS-FNRS, Rue d'Egémont 5 - 1000 Bruxelles, Belgium

## Background

Expansion of mesenchymal stem cells (MSC) is one of the key steps for their use in tissue engineering or cell therapies. Today, expansion processes are mainly based on the use of microcarriers to allow large interfacial adherence areas [1]. However, this culture technology is known to be practically limited to low agitation intensity and microcarrier concentrations due to possible cell damage arising from particle hydromechanical stress or collisions between microcarriers [2]. Unfortunately, the description of the relationship between bioreactor hydrodynamics, microcarrier suspension and occurrence of collisions was neither clearly established in the case of stem cell cultures, nor based on a local description of the bioreactor hydrodynamics heterogeneity. Thus, in the present study, it is proposed to use numerical simulations to describe not only the liquid phase but also the microcarrier dispersion and the occurrence of hydromechanical stress encountered by the microcarriers. Two kinds of hydromechanical stress can be distinguished: (i) fluid-solid interactions (fluid shear stress) arising from turbulent eddies and (ii) solid-solid interactions arising from collisions between microcarriers or between microcarriers and bioreactor walls [2].

## Materials and Methods

Computational Fluid Dynamics (CFD) was used to simulate the hydrodynamics inside a hemispherical-bottom bioreactor (working volume 1.12 L). Agitation was ensured using an axial HTPG or elephant ear (EE) impeller. Cytodex-1 microcarriers were considered in this study with a concentration varying between 1 and 10 gL-1. Simulations of microcarrier suspension were carried out with ANSYS Fluent 14.5. An experimental characterization of the microcarrier suspensions was also performed inorder to define the most adapted model parameters and to validate the simulations. The diameter and density of the Cytodex-1 microcarriers were obtained from microscopic pictures of both dry and wet particles (in 1% Phosphate Buffered Saline) and implemented in the model. Settling velocities at various concentrations were measured in order to define the drag function to implement in the multiphase model. The minimal agitation rate that leads to a complete suspension of microcarriers, Njs, was determined via the Zwietering methodology. The spatial distribution of microcarriers was visually observed for agitation rates below and above NJs to qualitatively determine the accuracy of CFD simulations. Finally, the Eulerian-Granular two-fluid approach was used in order to take into account the collisions between the particles. Turbulence was modelled with the dispersed k-ο formulation and the drag function was described by the Huilin-Gidaspow model. Simulations were performed using the Moving Reference Frame approach with agitation rates below and above the experimental Njs for both impellers.

## Results

**Figure 1:**
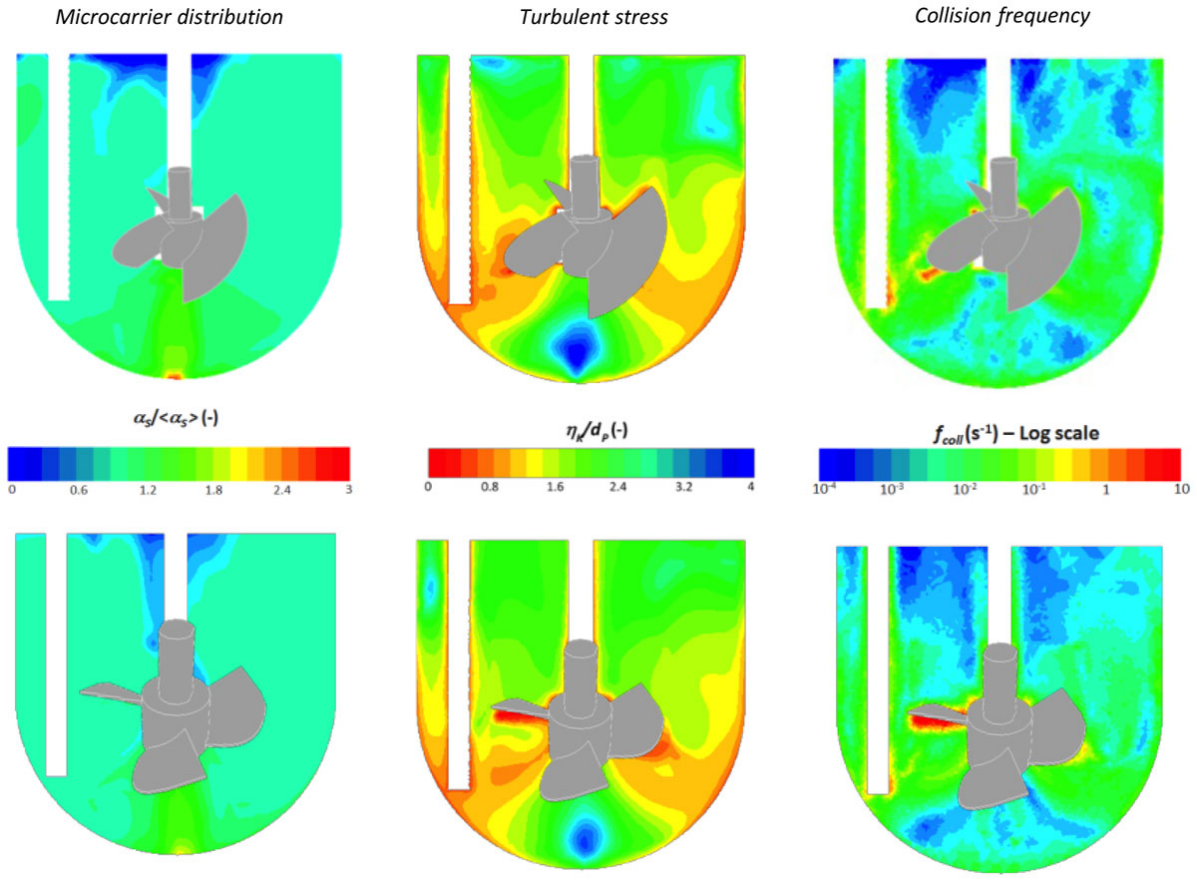
**Microcarrier distribution (left), ratio of Kolmogorov scale to particle diameter (middle) and collision frequency for the elephant ear (top figures) and HTPG (bottom figures) impellers, operating respectively at *N_js_*= 50 and 85 rpm (*P */ *V *= 1 W m^-3^) and a Cytodex-1 concentration of 10 g L^-1^**.

Simulations were qualitatively compared with visual observation of the microcarrier distribution inside the bioreactor for various agitation rates. They provided very satisfactory predictions of microcarrier suspension except at the bottom of the tank and near the free surface. Indeed, at agitation rates below Njs, an accumulation of unmoving particles was observed and a clear layer, free of particles, appeared below the liquid surface. These observations were well predicted by the model but for 10 rpm lower agitation rates compared to experiments. This leads to a slight underestimation of Njs for both impellers. Spatial distribution of microcarrier concentration and hydromechanical stresses encountered by microcarriers are compared between the two impellers at P / V = 1 W m-3 for a microcarrier concentration of 10 gL-1(Figure 1). The corresponding agitation rates were 85 and 50 rpm for the HTPG and the EE impeller, respectively (which was indeed equal to Njs for both impellers).The hydromechanical stress arising from the interaction between the liquid and the particles was characterized by the ratio of the Kolmogorov length scale to the particle diameter (λK/dP). The Kolmogorov scale corresponds to the size of the smallest eddies in a turbulent flow and are considered as the most likely to interact with small particles. The stress arising from the collisions between microcarriers or between the microcarriers and the bioreactor and impeller walls were related to the collision frequency [3].The two impellers promoted similar spatial distributions of microcarrier concentration and hydromechanical stress. However, the EE impeller exhibits slightly higher values of stress. As expected, the higher values of fluid shear stress and collision frequency were observed near the impeller blades.

## Conclusions and perspectives

A CFD numerical tool has been developed to model the solid-liquid suspension inside bioreactors used for expansion of MSC cells. Despite some slight approximations, the hydromechanical stress encountered by microcarriers inside stirred tank bioreactors could be estimated and compared between various bioreactor configurations. For the first time, the local stress related to the collisions of the microcarriers has been taken into account. To enhance the accuracy of the CFD simulations regarding the spatial distribution of microcarrier concentrations and the prediction of Njs, the influence of the particles on the fluid turbulence should be further studied. Aiming at this, diphasic PIV measurements will be carried out in the future.
